# Rare Lupus Exacerbation Shines Light on Provider Bias in a Patient With Possible Autoimmune Hepatitis

**DOI:** 10.7759/cureus.98015

**Published:** 2025-11-28

**Authors:** Sarah Petelinsek, Kasey Stoutin, Patrick Hughes, Alexander E Kolomaya

**Affiliations:** 1 School of Medicine, The University of Utah, Salt Lake City, USA; 2 Department of Internal Medicine, The University of Utah, Salt Lake City, USA; 3 Department of Emergency Medicine, The University of Utah, Salt Lake City, USA

**Keywords:** alcohol-related liver disease, alcohol use disorder (aud), autoimmune hepatitis with cirrhosis, diagnostic bias, sytemic lupus erythematosus, tumid lupus erythematosus, unconscious bias

## Abstract

A 42-year-old woman with a past medical history of alcohol use disorder, decompensated alcoholic cirrhosis, systemic lupus erythematosus (SLE), rheumatoid arthritis, and celiac disease presented to the emergency department (ED) for a four-day history of painful rash with worsening pruritus and acute kidney injury. After admission, dermatology, rheumatology, and nephrology teams were consulted, and a skin and renal biopsy were obtained, with results significant for lupus erythematosus tumidus (LET), lupus nephritis grade III (not active), and IgA nephritis, consistent with a systemic lupus erythematosus exacerbation. Despite the patient's history of autoimmune disease, she had previously never received a biopsy for autoimmune hepatitis (AIH) during her initial diagnosis of cirrhosis. During her admission, an autoimmune hepatitis panel was obtained with results suggestive of autoimmune hepatitis; however, a conclusive diagnosis requires a liver biopsy, which was not obtained inpatient. In patients with a strong history of alcohol use, anchoring on substance use as the sole driver of liver disease may be easy, but this case highlights the importance of keeping a wide differential and considering related illnesses, even in patients with pre-existing diagnoses.

## Introduction

Medical misdiagnoses are common, with rates varying from 10% to 15% of cases demonstrating diagnostic errors [1-4]. In general, patients with atypical presentations, complex or rare diseases, language barriers, low health literacy, and socioeconomic disadvantage, and those from historically underserved racial and ethnic groups are at an increased risk of experiencing misdiagnosis or delays in their diagnosis [5]. Specifically, patients with alcohol use disorder or other substance use disorders are at a high risk for misdiagnoses or delayed diagnoses, placing them at risk of increased morbidity or mortality [6-8]. The high rates of diagnostic error among these vulnerable populations suggest a greater need to rely on empirical evidence and explore possibilities of overlapping conditions.

The overall prevalence of autoimmune hepatitis (AIH) is estimated to be 31.2 per 100,000 patients [9]. Despite this low prevalence in the total population, between 14% and 44% of patients with autoimmune hepatitis have concurrent autoimmune diseases, which suggests an overlap of autoimmune hepatitis and other autoimmune diseases [9]. For this reason, the American College of Gastroenterology clinical guidelines strongly recommend that all patients with abnormal aspartate aminotransferase (AST) and alanine transaminase (ALT) levels, and particularly patients with autoimmune conditions, should be screened for autoimmune hepatitis (AIH) with anti-nuclear antibody (ANA), anti-smooth muscle antibody (SMA), and globulin level tests (IgG) [10]. These tests classically screen for AIH, while a formal diagnosis of autoimmune hepatitis requires histologic confirmation on liver biopsy [10]. The International Autoimmune Hepatitis Group has developed a diagnostic scoring system to aid in identifying patients with likely AIH and who may benefit from biopsy, especially when they have overlapping conditions [11,12].

Lupus erythematosus tumidus (LET) is a rare subtype of cutaneous lupus erythematosus that traditionally presents with a characteristic rash, without systemic symptoms. The prevalence of lupus erythematosus tumidus is not yet known, with the condition only being documented through case studies and series [13,14]. The typical rash in lupus erythematosus tumidus presents as smooth, erythematous, urticarial-like plaques. These plaques lack epidermal changes and thus are traditionally non-scarring. This also distinguishes the lupus erythematosus tumidus rash from other forms of cutaneous lupus erythematosus [13-16]. Histologically, lupus erythematosus tumidus is distinguished by a dense perivascular and periadnexal lymphocytic infiltrate in the dermis and significant mucin deposition. It lacks prominent interface dermatitis, and there is an absence of epidermal atrophy or alteration in the dermal-epidermal junction seen in other forms of cutaneous lupus erythematosus [13-16].

Distinguishing lupus erythematosus tumidus from other types of cutaneous lupus erythematosus is important because lupus erythematosus tumidus has a comparatively benign clinical course, and it generally responds to treatment with immunosuppressive medications and topical corticosteroids [13-16]. Appropriate recognition of lupus erythematosus tumidus prevents unnecessary exposure to systemic treatment. We present a case where a prior diagnosis of alcoholic cirrhosis is challenged during a hospitalization for systemic lupus erythematosus (SLE) flare, highlighting the complexities of comorbidity and cognitive bias potentially delaying appropriate diagnosis.

## Case presentation

The case being presented is that of a 42-year-old woman with a past medical history of alcohol use disorder, decompensated alcoholic cirrhosis, systemic lupus erythematosus, rheumatoid arthritis, and celiac disease who presented to the emergency department (ED) and was admitted for a four-day history of painful rash with worsening pruritus. Upon initial presentation, the patient's chief complaint was a diffuse, painful, pruritic, urticarial rash across her face, chest, and upper torso. The rash began the same day that the patient moved to a new home and started a new job; however, outside of the change of residence and her career, the patient had no other changes to her normal routine, including no new medications, vaccinations, or other exposures. The patient was up to date on all vaccinations. She reported variable alcohol use over the past decade, with greater than three months of sobriety (100 days at the time of admission) from alcohol at the time of admission. In addition to the rash, the patient was experiencing nausea with two episodes of vomiting, generalized myalgias, arthralgias, diffuse edema, and exertional dyspnea.

**Figure 1:**
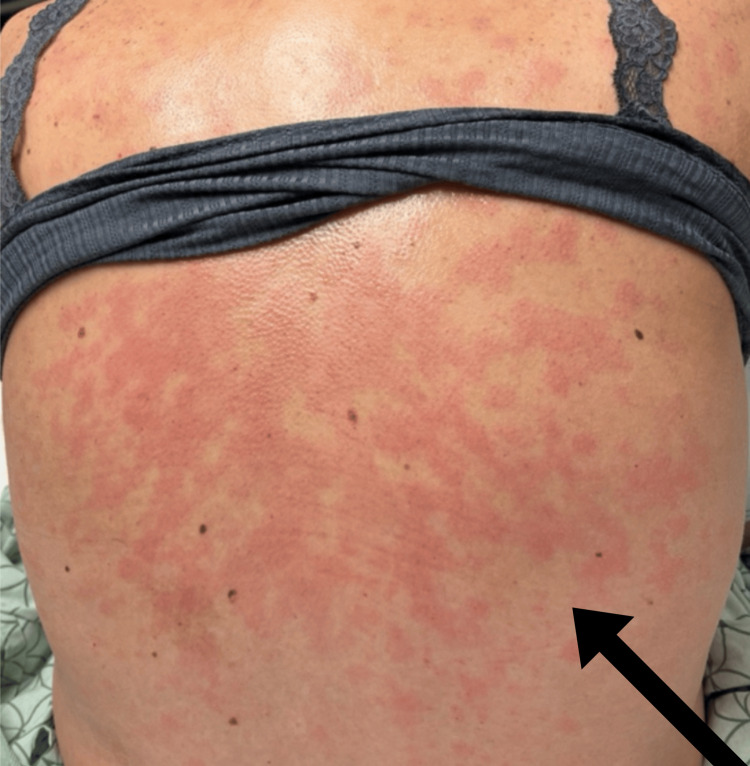
Photograph of the patient's diffuse, painful, pruritic, urticarial rash at presentation The arrow highlights the location of the rash. Later skin biopsy demonstrated interface dermatitis and lymphocytic periadnexal infiltrate, and increased mucin, results consistent with LET. This finding, in combination with the patient's other symptoms, demonstrates a case of systemic LET. LET: lupus erythematosus tumidus

The patient initially attributed the pruritus to a symptom of her decompensated alcoholic cirrhosis; however, after developing the painful rash, she decided to seek medical care. Her personal and family history were significant for multiple autoimmune and rheumatologic conditions, including Sjogren's disease and rheumatoid arthritis. On physical examination, she was found to have right upper quadrant rebound tenderness and bilateral lower extremity pitting edema in addition to a diffuse erythematous urticarial rash (Figure 1). Further workup was significant for acute kidney injury with proteinuria, hematuria, and pancytopenia (Table 1 and Table 2). Her blood alcohol level was undetectable.

**Table 1:** Patient key laboratory results (serum) This table includes the laboratory results obtained from the patient at the time of admission. Notable findings include pancytopenia and an increase in her creatinine from her baseline, suggestive of an acute kidney injury. The patient's blood alcohol at the time of admission was undetectable. Na: sodium, K: potassium, Cl: chloride, HCO3: bicarbonate, BUN: blood urea nitrogen, AST: aspartate aminotransferase, ALT: alanine transaminase, EGFR: epidermal growth factor receptor, ESR: erythrocyte sedimentation rate, WBC: white blood cell, RBC: red blood cell, MCV: mean corpuscular volume, MCHC: mean corpuscular hemoglobin concentration, hCG: human chorionic gonadotropin, INR: international normalized ratio

Variables	Results	Reference range	Units
Na	134	136-144	mmol/L
K	3.5	3.3-5.0	mmol/L
Cl	108	102-110	mmol/L
HCO3	18	20-26	mmol/L
BUN	19	8-24	mg/dL
Creatinine S/P	1.06	0.57-1.11	mg/dL
Glucose	94	64-128	mg/dL
Anion gap	8	5-14	mmol/L
Calcium	8.7	8.4-10.5	mg/dL
Protein, total, S/P	6.7	6.5-8.4	g/dL
Albumin	2.9	3.5-5.0	g/dL
Bilirubin, total	1.7	0.2-1.4	mg/dL
Alkaline phosphatase	120	38-126	U/L
AST	66	16-40	U/L
ALT	30	0-55	U/L
EGFR	67	-	mL/min/1.73 m^2^
ESR	30	<20	mm/hour
WBC	4.92	4.30-11.30	k/uL
Hemoglobin	9.9	12.6-15.9	g/dL
Hematocrit	29.7%	36%-49%	%
Platelets	100	159-439	k/uL
RBC	3.41	4.08-5.47	M/uL
MCV	87.1	81.9-101.0	fL
MCHC	29.0	25.8-33.1	pg
Red cell distribution width	16%	11.5%-15.3%	%
Mean platelet volume	10.7	8.6-12.3	fL
Neutrophil %	70.4%	39.4%-72.5%	%
Lymphocyte %	15%	17.6%-49.6%	%
Monocyte %	11%	4.1%-12.4%	%
D-dimer	1.0	0.0-0.4	ug/mL
Prothrombin time	17.1	12.0-15.5	Seconds
Beta-hCG, serum qualitative	Negative	-	-
Ethanol STAT	<10	≤10	mg/dL
INR	1.4	-	-

**Table 2:** Patient key laboratory results (urine) This table includes the laboratory results obtained from the patient's urine at the time of admission. These results were notable for proteinuria and hematuria. Further urinalysis revealed 3+ blood, RBCs, several dysmorphic RBCs, and some red blood cell casts, demonstrating kidney involvement in the setting of the patient's cutaneous lupus. RBC: red blood cell, WBC: white blood cell, EPI: epithelial cell, HPF: high-power field, LPF: low-power field

Variables	Results	Reference range	Units
Urine, color	Dark yellow	-	-
Appearance, urine	Cloudy	Clear	-
Specific gravity, urine	>1.050	1.003-1.030	-
pH, urine	5.5	5.5-7.5	-
Protein, urine	30	Negative	-
Glucose, urine	Negative	Negative	-
Ketones, urine	Trace	Negative	-
Bilirubin, urine	Negative	Negative	-
Blood, urine	Large	Negative	-
Leukocyte esterase, urine	Trace	Negative	-
Nitrites, urine	Negative	Negative	-
Urobilinogen, urine	1.0	<2.0	mg/dL
Bacteria, urine	Few	Negative	-
RBC auto, urine	325	0-5	/HPF
WBC auto, urine	10	0-5	/HPF
EPI auto, urine	22	0-5	/HPF
Casts auto, urine	<4	0-3	/LPF

Multiple diagnostic avenues were pursued after admission to the internal medicine service, dermatology, rheumatology, and nephrology teams were consulted. With the patient's rash, her urinalysis (UA) with 3+ blood and red blood cell casts, and elevated erythrocyte sedimentation rate (ESR), our principal concern was an exacerbation of SLE, which we pursued with a skin and renal biopsy. The skin biopsy demonstrated interface dermatitis and lymphocytic periadnexal infiltrate, and increased mucin, results consistent with lupus erythematosus tumidus. The renal biopsy demonstrated findings consistent with lupus nephritis grade III (not active) and IgA nephritis. Her rash and renal function improved after initiation of immunosuppression, but her peripheral edema remained persistent.

Her pancytopenia and elevated international normalized ratio (INR) were attributed to her cirrhosis, and during admission, it was noted that she had yet to have a comprehensive evaluation for autoimmune causes despite her comorbidities. An autoimmune hepatitis panel was also obtained, with low positive markers of autoimmune hepatitis with a positive ANA (1:320) and SMA (1:40). Initially, while her cirrhosis could be explained by prior alcohol use, these serologic tests would promote a liver biopsy under the diagnostic scoring system​​ (Table 3).

**Table 3:** Diagnostic scoring system for autoimmune hepatitis from the International Autoimmune Hepatitis Group A score of >15 represents a definitive diagnosis of autoimmune hepatitis, and a score between 10 and 15 represents a probable diagnosis of autoimmune hepatitis. Our patient scored a 13 without a biopsy, which is suggestive of a probable diagnosis of autoimmune hepatitis. AP: alkaline phosphatase, AST: aspartate aminotransferase, ANA: antinuclear antibody, SMA: smooth muscle antibody, anti-LKM1: anti-liver-kidney microsomal type 1, AMA: antimitochondrial antibody, HLA: human leukocyte antigen, LC1: liver cytosol type 1, pANCA: perinuclear antineutrophil cytoplasmic antibody

Category	Component	Scoring threshold	Our patient's results	Points	Our patient's points
Demographics	Sex	Female	Female	+3	+3
Biochemistry	AP:AST ratio	>3	97:63 (1.54)	-2	0
		<1.5	97:63 (1.54)	+2	0
Serology - immunoglobulins	IgG above normal	>2.0	1,553 (reference: 768-1632)	+3	0
		1.5-2.0	1,553	+2	0
		1.0-1.5	1,553	+1	0
		<1.0	1,553	0	0
Serology - autoantibodies	ANA, SMA, or anti-LKM1 titers	1:80	ANA: 1:320	+2	+2
		1:40	SMA: 1:40	+1	+1
		<1:40	LKM1: 1.2	0	0
	AMA	Positive	Negative	-4	0
Viral markers	Viral hepatitis serology	Positive	Negative	-3	0
		Negative	Negative	+3	+3
Exclusion factors	Drugs	Yes	No	-4	0
		No	No	+1	+1
	Alcohol	<25 g/day	Sober >100 days	+2	+2
		>60 g/day	Sober >100 days	-2	0
Genetic/immune	HLA	DR3 or DR4	Not tested	+1	0
	Immune disease	Thyroiditis, colitis, others	Present	+2	+2
	Other markers	Anti-SLA, actin, LC1, pANCA	Anti-SLA: negative, LC1/pANCA: not ordered	+2	0
Histology	Interface hepatitis	Present	No liver biopsy performed	+3	0
	Plasmacytic	Present	No liver biopsy performed	+1	0
	Rosettes	Present	No liver biopsy performed	+1	0
	None of the above	-	No liver biopsy performed	-5	0
	Biliary changes	-	No liver biopsy performed	-3	0
	Other features	-	No liver biopsy performed	-3	0
Treatment response	Complete	-	Not treated for AH	+2	0
	Relapse	-	Not treated for AH	+3	0

Ultimately, her renal and dermatologic manifestations remained the focus of her inpatient stay. However, her comprehensive inpatient evaluation resulted in a score of 13 on the diagnostic scoring system for autoimmune hepatitis from the International Autoimmune Hepatitis Group. A score of >15 represents a definitive diagnosis of autoimmune hepatitis, and a score between 10 and 15 represents a probable diagnosis of autoimmune hepatitis. She was referred back to hepatology for additional consideration of an outpatient liver biopsy. She was started on mycophenolic acid 500 mg BID and hydroxychloroquine 200 mg daily for treatment of her lupus flare until outpatient follow-up with rheumatology.

## Discussion

Our case underscores two important features about her presenting dermatologic symptoms: lupus erythematosus tumidus is a rare presentation of systemic lupus erythematosus that providers may not associate with a lupus flare. Additionally, other sources report the absence of systemic involvement in the setting of lupus erythematosus tumidus, with the primary symptom being the erythematous, edematous rash, with generally minimal other clinical features [13-16]. However, we demonstrate the potential for systemic symptoms (hematuria, proteinuria, kidney biopsy with focal lupus nephritis, and IgA nephropathy) in the setting of lupus erythematosus tumidus (as diagnosed by skin biopsy).

We also draw attention to a significant diagnostic challenge throughout our case. The presentations of alcoholic liver disease and autoimmune hepatitis often present similarly, which makes differentiating the diagnoses inherently challenging [17]. The American College of Gastroenterology recommends that patients with a history of autoimmune disease be evaluated for autoimmune hepatitis [10,17-20]. Although she was established with a hepatologist, and outside records demonstrated a strong suspicion for alcoholic cirrhosis, our patient had never previously received serologic studies or a liver biopsy. After serologic studies were performed and the diagnostic scoring system reached 13, meeting "probable" criteria for AIH, it was suggested that she may benefit from a liver biopsy to resolve the diagnostic uncertainty. The longest delay in her potential diagnosis may be attributable to anchoring bias, as multiple providers deferred to her hepatologist's diagnosis of alcoholic cirrhosis. Additional considerations for delay may be focused on two components of utility. First, the patient already had sequelae of decompensated cirrhosis with ascites often considered a clinical endpoint for preventative practice. Second, the utility of pursuing an AIH diagnosis given an already present indication for immunosuppressive treatment due to the patient's SLE. Her case was additionally complicated by intermittent compliance with immunosuppressive medications for systemic lupus erythematosus and side effects from previous trials of azathioprine and hydroxychloroquine.

Ultimately, this case highlights the complexities of multimorbidity and the importance of clinician awareness of cognitive biases during the diagnostic process. A systematic review by Saposnik et al. found that cognitive biases affect between 36.5% and 77% of case scenarios [21]. Approximately 70% of cognitive biases potentially contribute to diagnostic errors, the most prevalent being diagnostic bias, anchoring bias, availability bias, and confirmation bias [22]. We specifically draw attention to two of the most prevalent cognitive biases: anchoring bias and availability bias.

Anchoring bias, sometimes more informally referred to as diagnostic momentum, is when a person locks in on a specific fact or piece of information and uses that frame to set expectations for additional outcomes. The "anchor" in this case was the patient's known alcohol use disorder. This known diagnosis framed the perspective in which we initially investigated her hepatologic and pruritic symptoms, delaying the investigation of overlapping hepatologic conditions.

Availability bias refers to the tendency for people to believe information that is easier to recall, as opposed to information that is accurate. For example, after seeing a car accident, people are more likely to overestimate the risk of driving. For our patient, availability bias was likely a factor because at the time of her admission, we had several other patients on the service being treated for complications secondary to alcohol and substance use disorder. This likely resulted in us overestimating the likelihood of her hepatologic symptomology being secondary to alcoholic cirrhosis, as opposed to AIH.

These cognitive and implicit biases led to a delay in diagnosis and treatment for our patient. Our experience indicates the importance of continued dialogue focused on recognizing and minimizing cognitive biases, specifically anchoring bias and availability bias. This metacognitive process may be an effective method to minimize decision-making biases moving forward [23].

## Conclusions

This report demonstrates the importance of keeping a wide differential and considering related illnesses, especially in patients with pre-existing conditions. In patients with a strong history of alcohol use, anchoring on substance use as the sole driver of liver disease may be efficient; however, this decision-making bias can lead to misdiagnosis and delays in care.
